# Using DNA barcodes for assessing diversity in the family Hybotidae (Diptera, Empidoidea)

**DOI:** 10.3897/zookeys.365.6070

**Published:** 2013-12-30

**Authors:** Zoltán T. Nagy, Gontran Sonet, Jonas Mortelmans, Camille Vandewynkel, Patrick Grootaert

**Affiliations:** 1 Royal Belgian Institute of Natural Sciences, OD Taxonomy and Phylogeny (JEMU), Rue Vautierstraat 29, 1000 Brussels, Belgium; 2 Royal Belgian Institute of Natural Sciences, OD Taxonomy and Phylogeny (Entomology), Rue Vautierstraat 29, 1000 Brussels, Belgium; 3 Laboratoire des Sciences de l’eau et environnement, Faculté des Sciences et Techniques, Avenue Albert Thomas, 23, 87060 Limoges, France

**Keywords:** COI, cryptic species, DNA barcoding, geographic distances, taxonomy

## Abstract

Empidoidea is one of the largest extant lineages of flies, but phylogenetic relationships among species of this group are poorly investigated and global diversity remains scarcely assessed. In this context, one of the most enigmatic empidoid families is Hybotidae. Within the framework of a pilot study, we barcoded 339 specimens of Old World hybotids belonging to 164 species and 22 genera (plus two *Empis* as outgroups) and attempted to evaluate whether patterns of intra- and interspecific divergences match the current taxonomy. We used a large sampling of diverse Hybotidae. The material came from the Palaearctic (Belgium, France, Portugal and Russian Caucasus), the Afrotropic (Democratic Republic of the Congo) and the Oriental realms (Singapore and Thailand). Thereby, we optimized lab protocols for barcoding hybotids. Although DNA barcodes generally well distinguished recognized taxa, the study also revealed a number of unexpected phenomena: e.g., undescribed taxa found within morphologically very similar or identical specimens, especially when geographic distance was large; some morphologically distinct species showed no genetic divergence; or different pattern of intraspecific divergence between populations or closely related species. Using COI sequences and simple Neighbour-Joining tree reconstructions, the monophyly of many species- and genus-level taxa was well supported, but more inclusive taxonomical levels did not receive significant bootstrap support. We conclude that in hybotids DNA barcoding might be well used to identify species, when two main constraints are considered. First, incomplete barcoding libraries hinder efficient (correct) identification. Therefore, extra efforts are needed to increase the representation of hybotids in these databases. Second, the spatial scale of sampling has to be taken into account, and especially for widespread species or species complexes with unclear taxonomy, an integrative approach has to be used to clarify species boundaries and identities.

## Introduction

With over 11 400 described species, the superfamily Empidoidea represents one of the largest extant lineages of flies (Diptera, Brachycera) (Evenhuis et al. 2007, Pape et al. 2009). According to the most recent systematic revision (Sinclair and Cumming 2006), this superfamily comprises five families: the Atelestidae, Brachystomatidae, Dolichopodidae *sensu lato*, Empididae and Hybotidae. Commonly known as ‘dance flies’, the Empidoidea most likely originated in the Mesozoic (ca. 150 million years ago, Wiegmann et al. 2003) and now have a nearly cosmopolitan distribution. The high species diversity of this group is matched by high morphological diversity which is also very well expressed in genitalic traits. Genital morphology is still the main decisive diagnostic character used in the morphological identification and subsequent classification.

Studies carried out over the last few decades indicate the family Hybotidae is to be monophyletic (Chvala 1983, Collins and Wiegmann 2002, Sinclair and Cumming 2006, Moulton and Wiegmann 2007). The family includes ca. 2000 described species worldwide (Yang et al. 2007), and typically consists of small-bodied insects (i.e. 1–6 mm in total length). The vast majority of known hybotid species are predators that either hunt their prey in the air (e.g., some Ocydromiinae, Hybotinae) or on the ground (Tachydromiinae). These flies can be easily recognized by a spherical head with distinctive morphology as described by Sinclair and Cumming (2006), the presence of a palpifer, and fore-tibial gland, restriction of the gonocoxal apodeme to the anterolateral margin of the hypandrium, apex of antenna often with long, slender seta-like receptor, laterotergite bare, and R_4+5_ unbranched. Their male genitalia are also distinctive, and spectacular, the hypopygium being often rotated 45–90 ° to the right, so that the cerci, which are usually located on the dorsal side of the animal are now on the right side of the body. Sinclair and Cumming (2006) classified the Hybotidae into five subfamilies: the Hybotinae, Ocydromiinae, Oedaleinae, Tachydromiinae and Trichininae (the genus *Stuckenbergomyia* Smith, 1969 does not seem to fit into any of these and probably deserves its own subfamily). However, our current understanding of the phylogenetics, taxonomy and natural history of the Hybotidae is limited (Collins and Wiegmann 2002, Moulton and Wiegmann 2007), with several groups being little known (Sinclair and Cumming 2006). In addition, large parts of the distributional range of this family have been poorly explored (e.g. Central Africa, the Oriental region and Neotropics). In some of these regions the diversity of hybotid flies has probably been greatly under-estimated. For instance, Grootaert and Shamshev (2013) recently described 25 new hybotid species, all of which were collected during a short field expedition in the Democratic Republic of the Congo (D. R. Congo). The current study utilizes new tissue samples from specimens from a range of localities in Europe, Asia and Africa. It has been made possible by extensive field collections carried out by the senior author (P.G.), who has added substantially to material currently available from the Old World.

DNA barcoding (Hebert et al. 2003), based on a ca. 650 bp DNA region from the 5’ end of the mitochondrial COI gene, is an easy-to-use molecular approach that allows quick assignment of samples into ‘genetic’ groups. In situations where a reference database of specimen data and morphospecies identifications exists, this technique can be used for exploring and comparing species limits as defined by morphological vs. DNA-based criteria. Although the family Hybotidae is species-rich, genetic data (i.e., DNA barcode sequences) for this group are surprisingly scarce in public databases, such as GenBank and The Barcode of Life Data Systems – BOLD (Ratnasingham and Hebert 2007). While there are over 500 COI sequences of Empidoidea in GenBank, we could only find a single, correctly classified Hybotidae sequence. Furthermore, although there are several DNA barcoding projects underway in North America that are analyzing large numbers of Hybotid species, sequence data from these studies have yet to be made available to the public. We found four DNA barcode sequences of hybotids in BOLD, but these are from specimens collected in Canada, and therefore fall outside the geographical scope of our study, which is restricted to Old World taxa.

In the current paper, we optimized protocols for DNA barcoding of hybotid flies and performed a preliminary barcoding study on selected genera and species of this group. We hope that this approach will accelerate an inventory of hybotid flies. Here, we investigated the ability of the barcoding data coming from a large sampling of diverse Hybotidae to reveal cryptic species, patterns of geographic variation, and putative new species.

## Material and methods

A total of 339 specimens, representing 164 morphospecies of Hybotidae (see Supplementary file 1) were selected and sequenced for this study. All material was collected between 2008 and 2012 in three biogeographic realms: the Palaearctic (Belgium, Portugal, France and Russian Caucasus), Afrotropical (D. R. Congo) and Oriental (Singapore and Thailand) realms. Our outgroup taxon was *Empis tessellata* Fabricius, 1794 (Empidoidea, Empididae), of which two individuals were sequenced. Specimens were collected mainly using sweep nets and Malaise traps, and were initially preserved in 70% ethanol. After identification, all specimens were transferred to 96% ethanol and then stored at 4 °C in order to minimize DNA degradation. Either complete specimens or mid or hind legs were used for total genomic DNA extraction. Immediately prior to extraction, each tissue sample was placed in a microtube and air-dried. DNA extractions were carried out using the NucleoSpin Tissue kit (Macherey-Nagel). We followed the manufacturer’s instructions, but used a longer lysis time (i.e. around 24 hours). After lysis, fly specimens were transferred to absolute ethanol and were put back to the collection. Voucher specimens have been deposited in the entomological collections of the Royal Belgian Institute of Natural Sciences (RBINS).

PCR conditions were optimized by testing primer concentrations of 0.1, 0.2 and 0.4 µM and MgCl_2_ concentrations of 1.5–2 mM against a gradient of annealing temperatures. The best results were obtained by using the protocol as follows: each reaction (total volume of 25 µl) contained 2–3 µl DNA extract, 0.4 µM of each primer, 0.03 unit/µl Platinum Taq polymerase (Invitrogen), 1 × PCR Buffer, 0.2 mM dNTP, 2 mM MgCl_2_ and ca. 15 µl of sterile water. The COI region of interest was amplified using the standard animal barcoding primers, LCO1490 and HCO2198 (Folmer et al. 1994), and the primer pair TY-J-1460 and C1-N-2191 (Wells and Sperling 1999), with an annealing temperature of 45 °C and 48 °C, respectively, and 40 PCR cycles. PCR results were assessed using agarose gel electrophoresis and PCR products were purified on NucleoFast 96 PCR Plates (Macherey-Nagel). Sanger sequencing was carried out on an ABI 3130xl automated capillary sequencer using BigDye v1.1 or v3.1 chemistry (Life Technologies).

DNA sequences were checked and assembled with SeqScape v2.5 (Life Technologies). Neighbour-Joining (NJ) trees based on uncorrected (p) distances were calculated in MEGA5 (Tamura et al. 2011). Non-parametric bootstrapping with 1000 replicates was performed to evaluate branch support. Pairwise divergences at three levels (intraspecific, interspecific and intrageneric, as well as interspecific but not intrageneric) were calculated using R v2.15.2 and the *ape* package (Paradis et al. 2004).

## Results

Our sampling covered all the currently accepted subfamilies and tribes of the Hybotidae. At the generic level, we investigated 22 of the 66 known genera (see Table 1 for full details). The DNA sequence data set consisted of 341 COI sequences (339 Hybotidae and two Empididae), each sequence being of 657 bp in length. These sequences were deposited in BOLD and GenBank (BOLD Process IDs EMPID001-13–EMPID341-13).

**Table 1.** Global suprageneric systematics of Hybotidae (without the genus *Stuckenbergomyia*) and genera investigated in the current barcoding study.

Subfamily (Tribe)	Number of genera	Investigated genera
Trichininae	2	1 (*Trichina*)
Ocydromiinae	15	3 (*Leptopeza*, *Ocydromia*, *Oropezella*)
Oedaleinae	4	3 (*Allanthalia*, *Euthyneura*, *Oedalea*)
Tachydromiinae		
- Symballophthalmini	1	1 (*Symballophthalmus*)
- Tachydromiini	8	4 (*Ariasella*, *Platypalpus*, *Tachydromia*, *Tachypeza*)
- Drapetini	18	6 (*Chersodromia*, *Crossopalpus*, *Drapetis*, *Elaphropeza*, *Nanodromia*, *Stilpon*)
Hybotinae		
- Bicellariini	13	1 (*Bicellaria*)
- Hybotini	14	3 (*Hybos*, *Syndyas*, *Syneches*)

**Figure 1.**
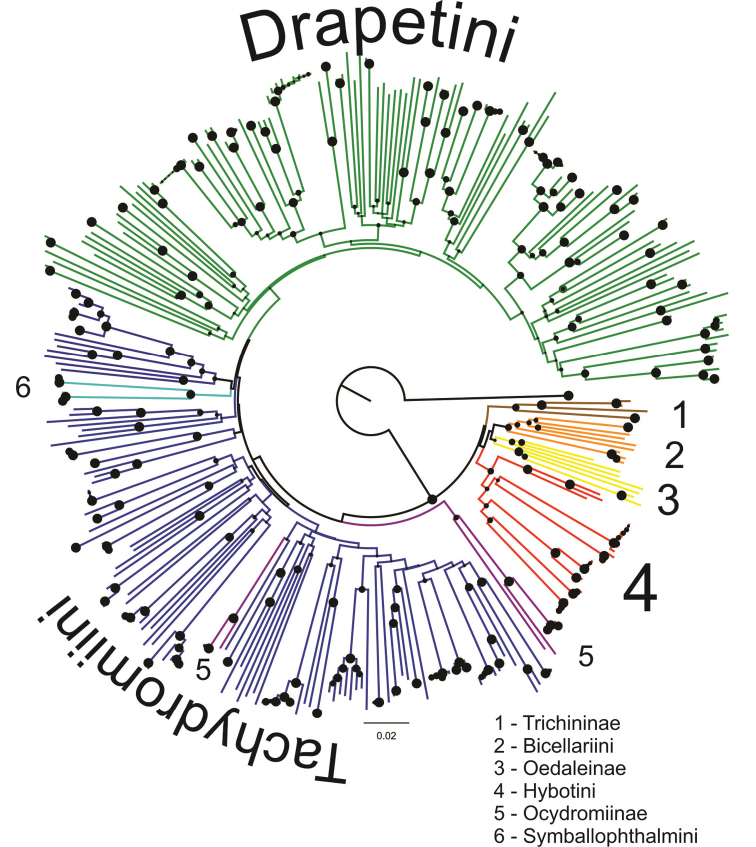
Neighbour-Joining tree representing hybotid diversity of 339 selected samples. The tree was rooted with *Empis tessellata* (Empididae). Circles represent branch supports, bootstrap values are according to circles’ size.

An NJ tree without species names and additional sample information is shown in Figure 1 (a fully annotated tree is shown in Supplementary file 2). ‘Species-level’ groups (i.e., close to or at terminal nodes) were generally well supported, while deep-level groups were not (see Figure 1 and Supplementary file 2). Especially when low intraspecific distance was observed, these groups (considered as molecular operational taxonomic units – MOTUs) often received 100% bootstrap support. At a 1% distance threshold (as it is also used by BOLD), 99% of the clusters (i.e., 70 out of 71 MOTUs) were supported by bootstrap values above 95%. Many recognized species were well resolved and distinguished using the COI data, but we observed a number of problems that are discussed below. Although representatives of more inclusive taxa, such as tribes and subfamilies (with the exception of the Ocydromiinae and Tachydromiini), were usually recovered in single clusters, these clusters were not supported (bootstrap values < 70%). The only exception is the tribe Symballophthalmini, represented in the data set by just two species, which was supported by a bootstrap value of 87.7%.

For most genera, the number of species represented in our analysis was very limited. Similarly, the number of conspecific sequences was also generally low, ranging between 1–9. Nonetheless, we observed considerable overlaps between intraspecific (0–17.2%) and interspecific divergences (0–21.81%). Among congeners, interspecific divergences ranged between 0–19.9%, while we observed higher divergence between samples of different genera (5.85–21.81%). Hence, no barcoding gaps existed between any of these ranks. The ranges of pairwise distances were overall high in the four genera represented by the highest number of samples (Table 2). We observed extensive overlap between intra- and interspecific divergences in both the species-rich genera of Tachydromiini, *Platypalpus* and *Tachydromia*, with less extensive, or no overlap, in the genera *Chersodromia* and *Elaphropeza*, both belonging to the Drapetini (Table 2).

Below, we describe five categories of cases where ranges of intra- and interspecific distances did not seem consistent with the current taxonomy and would require more investigation.

**Table 2.** Patterns of intra- and interspecific distances observed in four species-rich genera of our dataset.

Tribe	Genus	No. of species	No. of sequences	No. of haplotypes	Intraspecific distances (%)	Interspecific distances (%)
Tachydromiini	*Platypalpus*	45	98	81	0–16.89	0–18.72
Tachydromiini	*Tachydromia*	12	21	18	0–5.48	1.07–18.11
Drapetini	*Chersodromia*	12	36	26	0–3.04	6.09–15.53
Drapetini	*Elaphropeza*	43	75	68	0–5.48	1.83–19.63

### Different patterns of intraspecific divergence in congeneric species

**Figure 2.**
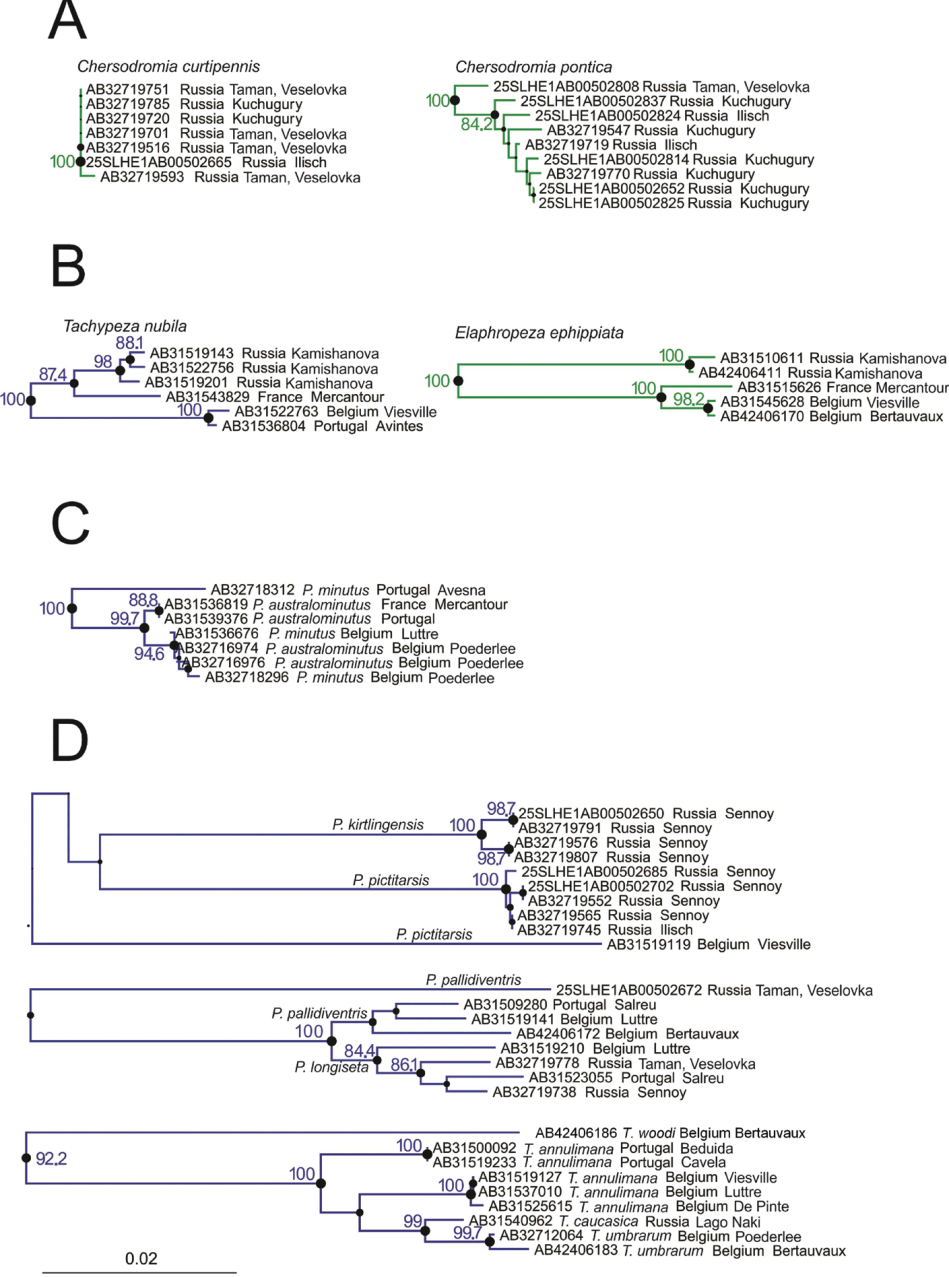
Subtrees showing cases where ranges of intra- and interspecific distances do not seem consistent with the current taxonomy and would require more investigation. See details in text. Circles represent branch supports. Bootstrap values are according to circles’ size, bootstrap values are shown in numbers when > 80%.

We found that in some congeneric species the levels of sequence variation observed both within populations and between populations in close proximity were low. Contrastingly, other congeneric species showed widely different levels of intraspecific divergence. An interesting case in this respect is the brachypterous *Chersodromia curtipennis* and the fully-winged *Chersodromia pontica*, both of which occur on the Taman Peninsula (Krasnodar region of Russia). Samples were taken at various sites on the Taman Peninsula, ranging from the North, on the coast of the Sea of Azov, to the South along the Black Sea (Taman: Veselovka). While the brachypterous species showed virtually no genetic variation (uncorrected pairwise divergence was between 0–0.15%), the fully-winged species showed an expressed pattern of divergence with pairwise p-distances of 0–1.37% (Figure 2A).

### Variation in intraspecific divergence may be related to spatial distance

Some species sampled across large geographical areas showed high levels of genetic divergence between populations. This is among others the case for two species that are widespread and very common in Europe: *Tachypeza nubila* and *Elaphropeza ephippiata*. While we could not detect any morphological differences (i.e. of the body and the male genitalia) between populations in western European and the Russian Caucasus, intraspecific pairwise genetic divergences ranged between 0.3–3.5% in *Tachypeza nubila* and between 0.2–5.48% in *Elaphropeza ephippiata* (Figure 2B). Unexpectedly large ‘intraspecific’ divergences may indicate undescribed diversity at the species level. In many cases, large ‘intraspecific’ divergences were found between specimens from the same locality or from adjacent sites (Table 3, upper part), and examples in this respect include *Platypalpus caucasicus* (Russian Caucasus), *Platypalpus annulipes* (Belgium), *Trichina elongata* (Russian Caucasus), *Bicellaria nigra* (Russian Caucasus), *Tachydromia annulimana* (within Europe), *Elaphropeza nuda* (D. R. Congo) and *Elaphropeza monospina* (Singapore). In a number of other cases, large ‘intraspecific’ divergences were observed between geographically distant populations (Table 3, lower part); this was observed for *Platypalpus pictitarsis* (Russian Caucasus versus Belgium), *Platypalpus pallidiventris* (Russian Caucasus versus Europe), *Leptopeza flavipes* (Russian Caucasus versus Belgium), *Oedalea zetterstedti* (Russian Caucasus versus Belgium), *Euthyneura myrtilli* (Russian Caucasus versus Europe), *Platypalpus nigritarsis* (Russian Caucasus versus France) and *Tachydromia aemula* (Russian Caucasus versus Portugal). In all of these cases, morphological differences of genitalia (or other diagnostic characters) were not assessed in details, and therefore these divergences may well reflect interspecific differences. Remarkably, no significant differences in divergence ranges were observed between the two types of cases (i.e., associated or not with large spatial distances).

**Table 3.** Range of pairwise p-distances in cases where unexpectedly high ‘intraspecific’ divergence was observed (> 5%).

Species or species complex	Range of pairwise p-distances (%)
*Platypalpus caucasicus*	0.46–8.07
*Platypalpus annulipes*	0–9.80
*Trichina elongata*	0.91–8.83
*Bicellaria nigra*	9.44
*Tachydromia annulimana*	0–10.35
*Elaphropeza nuda*	0–5.33
*Elaphropeza monospina*	5.33
*Platypalpus pictitarsis*	0–10.20
*Platypalpus pallidiventris*	1.37–10.05
*Leptopeza flavipes*	0–7.01
*Oedalea zetterstedti*	7.91
*Euthyneura myrtilli*	1.52–10.96
*Platypalpus nigritarsis*	5.33
*Tachydromia aemula*	5.48

### Genetic overlap of putative ‘sister’ species

*Platypalpus minutus* and *Platypalpus australominutus* are externally very similar except that male genitalia are consistently different (Grootaert 1989): In northern Belgium both species are sympatric and often occur syntopically. More to the South of Belgium and in the South of France mainly *Platypalpus australominutus* occurs. The Belgian specimens of these two species could not be distinguished by COI sequences due to shared haplotypes (Figure 2C). However, a specimen from Portugal provisionally identified as *Platypalpus minutus* was quite different from the clade *australominutus*-*minutus* from Belgium.

### Complex taxonomy

In Figure 2D, three examples are shown where the unclear taxonomy of the involved species or species complex was reflected in para- or polyphyletic taxa. For example, *Platypalpus pictitarsis* and *Platypalpus kirtlingensis* are morphologically very similar. They differ in the colour of the fore leg and the palpus. Barcode sequences showed that both species are genetically different (uncorrected pairwise interspecific divergence was at least 8.37%). In addition, however, a single male of *Platypalpus pictitarsis* from Belgium (AB31519119) rendered this taxon polyphyletic. Both external morphology and genitalia of the Belgian and Caucasian *pictitarsis* was the same and a deeper study will be needed to clarify this issue.

Another example involved the sister species *Platypalpus pallidiventris* and *Platypalpus longiseta*, which differ morphologically only in a few but distinct characters. Also, these species were genetically closely related except a single specimen of *Platypalpus pallidiventris* (25SLHE1AB00502672, see Figure 2D), which rendered this species paraphyletic. This single specimen of *Platypalpus pallidiventris* from Caucasus is a female, exhibiting less diagnostic characters than males, and could therefore belong to another species. This observation urges for a more intensive collection and study of these sister species. When we discarded this divergent sequence, both species showed a moderate intraspecific structuring. The bootstrap value supporting the cluster containing both species without the divergent specimen was 100%. In addition, the reciprocal monophyly of both species was supported with bootstrap values of 77.3% and 84.4% for *Platypalpus pallidiventris* and *Platypalpus longiseta*, respectively.

A third example involved four species (Figure 2D). Originally, a female (AB42406186) of *Tachydromia woodi* was identified as *Tachydromia annulimana*. However, the considerable divergence at COI between this specimen and all other specimens of *Tachydromia annulimana* (10.35%) suggested a misidentification. A reexamination of the specimen revealed that *Tachydromia woodi* has the costa between vein R_1_ and R_2+3_ thickened, an unpublished feature that confirmed the misidentification. *Tachydromia caucasica* from Caucasus and *Tachydromia umbrarum* from Belgium, both also belonging to the *annulimana*-group, showed a very low interspecific divergence (1.07-1.52%). This suggests, in combination with the little morphological differences reported between the two species, a very close relationship between the two species and does not exclude that they are conspecific.

## Discussion

The barcoding of dipterans commenced relatively early as part of the global DNA barcoding initiative. On the one hand, DNA barcoding performed well in several lineages. However, so far mostly Holarctic dipterans have been investigated where species diversity is overall lower than in tropical biomes. For instance, DNA barcoding proved to work well for Canadian (Cywinska et al. 2006) and Chinese mosquitoes (Wang et al. 2012), Nearctic simuliids (Rivera and Currie 2009) and muscids (Renaud et al. 2012) and so on, but this approach was less extensively tested on tropical taxa. On the other hand, the usefulness of dipteran DNA barcodes in species identification has been criticized (e.g., Meier et al. 2006, Whitworth et al. 2007) and new criteria for specific assignment have been introduced (Meier et al. 2006, best match and best close match criteria). An inherent problem with dipterans is the possibly high amount of unknown diversity on a global scale leading to incomplete databases, the substantial age of some large radiations that is linked to (very) high mitochondrial sequence diversity, and the limited taxonomic expertize on particular groups hampering successful identification. Unfortunately, reference barcode libraries of species-rich taxa are often incomplete. In fact, in many insect groups a few common species are overwhelmed by a high number of rare species (Lim et al. 2011). While common species are likely better represented, rare species are often missing in barcode libraries. This may lead to imbalanced taxon representation. Another critical issue of DNA barcoding is the effect of geographical sampling (Bergsten et al. 2012). Generally, identification success is dropping with increasing spatial scale of sampling, and may pose a real problem for all widespread taxa. In summary, all of these issues make DNA barcoding difficult. Nevertheless, DNA barcoding has been generally advocated as a pragmatic first step in the integrative taxonomic framework, also for problematic taxa (Tan et al. 2010, Nagy et al. 2012).

In the meantime, several dipteran barcoding projects have been started, particularly with respect to medically, forensically or commercially (e.g., related to agriculture) relevant lineages such as mosquitoes, muscids, tephritids and drosophilids (see details at http://boldsystems.org). Hybotids, or more broadly the empidoids, have no known medical, forensic or commercial importance, therefore there are overall much less intensively studied. The current dataset presented herein is a result of a pilot study focusing on Old World hybotid diversity. An overall high sequence divergence was observed in our dataset, which is not surprising in the light of the age and diversification pattern of dance flies (Wiegmann et al. 2003). Although most species could be well distinguished based on a single mitochondrial marker (Figure 1 and Supplementary file 2), we observed several inconsistencies with current classification and extensive overlaps of intra- and interspecific divergences.

Our limited sampling of specific and subspecific levels with up to nine samples per species did not allow us to perform extensive tests on barcoding performance and species (or genus) delimitations. We are also aware of the potential pitfalls when analyzing taxonomically incomplete datasets. In such cases, a hierarchical sampling should be followed whenever possible. In these cases, the number of sampled genera and more inclusive taxa should be maximized (Zhang et al. 2013). Here, we sampled all subfamilies and tribes, as well as one third of all hybotid genera, but sampling at the specific level remained far below 10%. Simulation of the sampling effect can be performed (e.g., Nagy et al. 2012), and this simulation may give hints about the power of DNA barcoding. Regarding species delimitation, simple methods relying solely on genetic distances are still broadly used, although there are many inherent problems with them (e.g., Meier et al. 2006). First, species delimitation simply based on genetic divergence is difficult to convey and interpret in a “universally acceptable” species concept (Krishnamurthy and Francis 2012). Second, large intraspecific distances and low interspecific distances among closely related species may pose a major problem, and even the use of refined criteria such as best close match (Meier et al. 2006) or *ad hoc* thresholds (Virgilio et al. 2012) might not solve this issue. Therefore, in datasets where intra- and interspecific divergences largely overlap, using distance-based thresholds alone may not work. In these cases, species or species complexes may have to be analyzed individually and also other DNA markers (including nuclear markers) should be considered for species delimitation and perhaps for revising our ideas about species identification.

In the case of recently diverged species, a number of methods have been compared (van Velzen et al. 2012) such as tree-based (Neighbour-Joining or tree-based parsimony), similarity-based (nearest neighbour or BLAST), statistical and diagnostic or character-based (e.g., BLOG: Bertolazzi et al. 2009, DNA-BAR: DasGupta et al. 2005) approaches. Similarity- and character-based methods have been shown to usually outperform tree-based methods (van Velzen et al. 2012), and some studies have found that character-based approaches may work better than distance-based methods (e.g. Bergmann et al. 2013). However, further analytical approaches need to be explored. Irrespective of the approach used, success in species identification can decrease with increasing sampling (Bergsten et al. 2012). Overall, the use of multi-gene markers and coalescent methods seem to be inevitable for efficient species delimitation (see Jörger et al. 2012), but this is clearly beyond the scope of DNA barcoding *sensu stricto.*

Although we focused on problematic or unexpected cases, in most of these examples, DNA barcoding may still be useful, provided that precautions are taken with respect to taxonomic and geographic sampling effects. Moreover, species identification in Hybotidae is based primarily on male terminalia and possibly some of the species concept situations are due to misidentification of females. Also, collecting precise information on collection site, life history, habitat, morphology etc. can very well contribute to the interpretation of the DNA barcoding results. Our finding about intraspecific divergence patterns in the brachypterous vs. the fully-winged species (*Chersodromia curtipennis* and *Chersodromia pontica*, respectively) exemplifies this well. The reduced mobility of the brachypterous species is apparently linked to the low intraspecific diversity but mechanisms are still unclear. In many cases where we found unexpectedly large intraspecific divergences (Table 3), we probably deal with undescribed species, and therefore, in fact, with interspecific divergences. Nevertheless, further investigations are necessary to clarify the taxonomic status of the divergent populations. We advocate in-depth investigations involving more diagnostic traits and multi-gene analyses, evaluated in an integrative taxonomic framework (Padial et al. 2010), even if these analyses may take longer time, and cost more (e.g., additional lab work needed to obtain further sequence data).

## Conclusions

In the current study, we provided a baseline for further studies on hybotid diversity using a DNA barcoding approach. We provided an optimized lab protocol for routine barcoding. We conclude that DNA barcoding can assist to identify hybotid taxa. Also cryptic species may be revealed by appropriate genetic markers, mostly because the morphological differences are not well assessed. Nevertheless, we emphasize to have an integrative look on barcoding data, and use this approach as a pragmatic first step in taxonomic practice or for biodiversity assessments.

## Appendix 1

Samples used in the current study. (doi: 10.3897/zookeys.365.6070.app1) File format: Microsoft Excel file (xls).

## Appendix 2

Neighbour-Joining tree representing hybotid diversity of 339 selected samples. (doi: 10.3897/zookeys.365.6070.app2) File format: Adobe PDF (pdf).

**Explanation note:** Neighbour-Joining tree representing hybotid diversity of 339 selected samples. The tree was rooted with *Empis tessellata* (Empididae). Bootstrap support was estimated with 1000 replicates.
