# Supplementary material for: Using DNA barcodes for assessing diversity in the family Hybotidae (Diptera, Empidoidea)
Source: Zookeys. 2013 Dec 30;(365):263–78. doi: 10.3897/zookeys.365.6070 (PMC3890682; doi:10.3897/zookeys.365.6070)

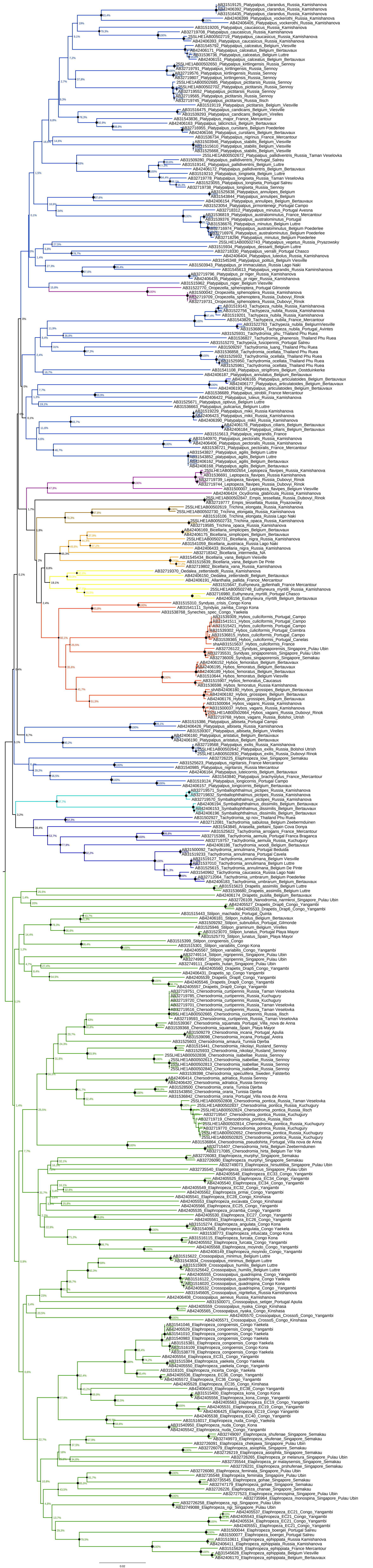

Supplement: Supplementary file 2 — Neighbour-Joining tree representing hybotid diversity of 339 selected samples. (doi: 10.3897/zookeys.365.6070.app2) File format: Adobe PDF (pdf). [file ZooKeys-365-263-s002.pdf]
